# Optical coherence tomography of the macular ganglion cell layer in children with neurofibromatosis type 1 is a useful tool in the assessment for optic pathway gliomas

**DOI:** 10.1371/journal.pone.0305548

**Published:** 2024-07-11

**Authors:** Urszula Arnljots, Maria Nilsson, Roberto Bolzani, Mariagrazia Benassi, Ulrika Sandvik, Daniel Martin Munoz, Klas Blomgren, Kerstin Hellgren

**Affiliations:** 1 Department of Clinical Neuroscience, Karolinska Institutet, Stockholm, Sweden; 2 St Erik Eye Hospital, Stockholm, Sweden; 3 Department of Psychology, University of Bologna, Bologna, Italy; 4 Department of Neurosurgery, Karolinska University Hospital Solna, Stockholm, Sweden; 5 Department of Neuroradiology and Pediatric Radiology, Karolinska University Hospital Solna, Stockholm, Sweden; 6 Pediatric Oncology, Astrid Lindgren Children’s Hospital Karolinska University Hospital, Stockholm, Sweden; 7 Department of Women’s and Children’s Health, Karolinska Institutet, Stockholm, Sweden; 8 Astrid Lindgren Children’s Hospital, Karolinska University Hospital, Stockholm, Sweden; Irrua Specialist Teaching Hospital, NIGERIA

## Abstract

**Background:**

Optic pathway glioma (OPG) is a feared complication to neurofibromatosis type 1 (NF1) since it can cause visual impairment in young children. The main goal of screening is to detect symptomatic OPGs that require treatment. Optical coherence tomography (OCT) has been suggested as a tool for detection of neuro-retinal damage.

**Aims:**

To investigate whether the ganglion cell layer assessed by OCT is a reliable measure to identify and detect relapses of symptomatic OPGs in children with NF1.

**Methods:**

Children (3–6 years) with NF1, with and without known OPG and children with sporadic OPG (S-OPG) resident in the Stockholm area, were invited and followed in a prospective study during a three-year period. Brain magnetic resonance tomography (MRI) had been performed in children with symptoms of OPG. Outcome measures were VA in logMAR, visual field index (VFI), average thicknesses of the ganglion cell-inner plexiform layer (GC-IPL), and peripapillary retinal nerve fiber layer (pRNFL).

**Results:**

There were 25 children with MRI-verified OPG and 52 with NF1 without symptomatic OPG. Eyes from NF1 patients without symptoms of OPG showed significantly better results in all four analyzed parameters compared to eyes with NF1-associated OPG. Mean GC-IPL measurements seemed stable and reliable, significantly correlated to pRNFL (correlation coefficient (r) = 0.662, confidence interval (CI) = .507 to .773 p<0.001), VA (r = -0.661, CI = -7.45 to -.551, p<0.001) and VFI (r = 0.644, CI = .452 to .774, p<0.001). GC-IPL measurements were easy to obtain and acquired at considerably younger age than pRNFL (5.6±1.5 vs 6.8±1.3; p<0.001).

**Conclusions:**

The mean GC-IPL thickness could distinguish well between eyes with OPG and eyes without symptomatic OPG in children with NF1. As thinning of GC-IPL assessed with OCT could indicate underlying OPG, it should be included in the screening protocol of children with questionable VA measurements and in particular in children with NF1.

## Introduction

It is well documented that the congenital disorder neurofibromatosis type 1 (NF1) entails an increased risk of developing tumors in the central and peripheral nerve system [1,2]. Low-grade astrocytic tumors occurring in the optic nerve, chiasm, optic tract, or optic radiations and are known as optic pathway gliomas (OPG). About 15% of children with NF1 develop OPG and the signs and symptoms of OPG are often evolving over months to years [3–6]. Ophthalmological symptoms are often the first signs of the OPG [7]. However, since ophthalmological symptoms may vary–from isolated discrete eye movement restrictions, papilledema to visual field (VF) defects, severe vision loss and blindness—early and adequate diagnosis remains a challenge in clinical settings. Even if visual acuity (VA) is assessed regularly, visual impairment (VI) can be misinterpreted as behavioral issues particularly in this group of children due to higher prevalence of developmental delay, specific learning problems, attention deficit hyperactivity disorder or autism spectrum disorders [8]. Severe delay is a challenge in children with OPG, even in those undergoing a screening program, and they often exhibit symptoms of varying duration indicating disease before diagnosis or detection of relapses. The diagnostic delay is stressful and may lead to an increased risk of visual loss as well as neurological and cognitive sequelae. Therefore, there is a need to include objective examination methods in the ophthalmological screening program of children with NF1. Ideally, the examination methods should be useful at young ages for early detection and intervention.

Optical coherence tomography (OCT) is a patient-friendly imaging technique, that provides cross-sectional images of all layers of the retina. Since a centrally caused axon injury, such as in the case of an OPG, causes a retrograde degeneration of the nerve fiber layer in the retina, the neural tissue loss can be detected and quantified with this high-resolution technique. The technique enables cross sectional visualization of the optic nerve head and macula. Measurements of the peripapillary retinal nerve fiber layer (pRNFL), and macular ganglion cell—inner plexiform layer (GC-IPL) can serve as an estimate of the afferent visual pathway structure and be used for detection and monitoring of the degree and progression of the degeneration [9].

A study by *Sahinoglu-Keskek et al*. found significantly lower pRNFL and ganglion cell complex thickness in NF1 patients with OPG than patients without OPG (NF1 and controls) (p < 0.001) [10]. Similar findings were reported by *Topcu-Yilmas et al*. who found, that pRNFL thickness was significantly thinner in patients with OPG compared to the control group as well as NF1 patients without OPG (p < 0.001). They also observed, that mean pRNFL was significantly thinner in patients with OPG irrespective of VA in comparison to healthy controls without NF1 [11]. To date, mainly pRNFL has been examined as a structural marker of visual integrity in patients with OPG [12,13]. There are only a few studies that discuss GC-IPL value among patients with OPG [14–16]. Two of them aiming at evaluating diagnostic accuracy in discriminating normal and abnormal visual function (VA/VF) using pRNFL and GC-IPL [14,15]. Another study determined the intra- and inter- reproducibility of GC-IPL thickness measurements in sedated children in two imaging sessions [16]. A limitation with the use of OCT in children is the lack of normative data in this age group. The reference values used in the manufacturer’s software are based on adults 18 years and older. Therefore, in a previous study we have established a normative pediatric reference range of the GC-IPL thickness [17]. Furthermore, in a subsequent study we have documented that GC-IPL thinning was associated with vision loss in children with OPG and that OCT therefore appeared to be a potential link between magnetic resonance imaging (MRI) and visual function tests [18]. This study has two aims. First, we wanted to compare ophthalmological findings, in particular GC-IPL, in children with MRI-verified OPG with children with NF1 but without symptoms of OPG. Second, we wanted to follow these children over a three-year period. To the best of our knowledge, there are no longitudinal studies describing how structural OCT can be used as a targeted tool to objectively measure clinical progression of ganglion cell loss in children with OPG. Therefore, this study aims to further investigate the reliability and prognostic value of OCT during a three-year follow-up of children with known OPG or at risk of developing OPG.

## Material and methods

### Patients

Children (3–6 years) with NF1 without symptoms suggestive of OPG, children with NF1 and MRI-verified OPG as well as children with sporadic OPG (S-OPG) were consecutively invited and followed in a prospective study at Astrid Lindgren Children’s Hospital during a three-year period (first of January 2017- first of January 2020). All children were resident in the Stockholm area. Brain MRI was performed only in the presence of clinical symptoms suggestive of OPG. Children with NF1 without symptomatic OPG were followed according to a screening program prepared for the study purposes. The follow-up intervals for this group were as follows: every three months for children three to four years old, every six months for children five to six years old and yearly in children older than seven years. In the group with symptomatic and documented OPG the frequency of ophthalmological assessments depended on the degree of VI and the associated symptoms, as well as the MRI findings. Written informed consent from the parents/custodians was obtained before study enrollment. Ethical approval was obtained (2016/1749-31/4).

A retrospective review was performed on all patients with OPG followed/treated in the Pediatric Oncology and/or Pediatric Neurosurgical Department at Astrid Lindgren’s Childrens’ Hospital at Karolinska University Hospital in Stockholm from first of January 2009 until first of January 2020 (study end). Patient data were obtained from medical documentation: clinical manifestation at the time of diagnosis (VI, neurological and/or endocrinological symptoms), age at OPG diagnosis, MRI findings (initial location and extension of the tumor as well as radiologic progression or regression), criteria to start treatment (visual decline or other), treatment regime (surgery, chemotherapy) as well as age at treatments and additional information such as cognitive deficit, specific learning problems, attention deficit hyperactivity disorder or autism spectrum disorders among patients with NF1.

### Magnetic resonance imaging

Retrospective analyses of OPG localization at the time of diagnosis were performed as previously described [18]. Thereafter, baseline MRI images were compared with all subsequent MRI images (from diagnosis to the end of the study) to see whether changes (progression or regression) resulted in treatment changes. The changes on MRI were recorded as progression or regression. Of those who performed 1.5T, 40% repeated the examination at 3T (the rest repeated at 1.5T). Of those who performed 3T, 60% repeated the emanation at 3T while the rest were followed with 1.5T.

### Groups

Eyes with age-normal VA and no VF defects were considered having no symptoms suggestive of OPG and thus the child did not undergo MRI, if no other clinical symptom (not ophthalmological) suggested otherwise. Three groups of eyes were determined: 1) Group “NF1 noOPG”; eyes without symptoms suggestive of OPG in patients with NF1. This group included both eyes from asymptomatic patients screened for OPG without having undergone MRI as well as the unaffected eye from NF1 patients with MRI verified unilateral OPG spread limited to the contralateral optic nerve. 2) Group “NF1-OPG”; eyes with MRI-verified NF1-associated OPG and 3) group “S-OPG”; eyes with MRI-verified sporadic OPG. Subjects underwent age-appropriate VA assessments, neuro-retinal measurements using OCT and automated VF examination. Outcome measures were VA, average GC-IPL, average pRNFL thicknesses and visual field index (VFI). The tumor-free eye from the cases with sporadic OPG, that is not NF1, were excluded from analyses.

### Visual acuity measurement

Monocular VA was quantitively measured at as a young age as possible. In the youngest group VA was assessed with Teller acuity cards or Cardiff acuity cards [19,20] while older children were tested with Lea or KM (Konstantin Moutakis) cards [21,22]. Visual acuity was reported as logMAR units (higher logMAR values corresponding to lower VA). Maximum VA on the chart was -0.3. Prespecified values were used to describe qualitative VA: counting fingers (CF = 2.0), hand movement (HM = 2.5), light perception (LP = 3.0) and no light perception (NLP = 3.5) as previously shown [23]. In children ≥4 years of age VA at initial visit was compared with World Health Organization Categories of Childhood VI scale for reference. In children <4 years of age, mild VI was not defined but moderate VI was defined as > 0.5 VA = < 1.0 and severe VI as >1.0 VA = <1.7, in accordance to our previous study [18].

### HD-OCT procedure

Optical coherence tomography images were obtained using Cirrus HD-OCT device (Cirrus; Carl Zeiss Meditec, Dublin, CA, USA). Cirrus uses ganglion cell + inner plexiform layer (GC-IPL) to assess thickness of Ganglion Cell Layer therefore GC-IPL never gets thinner than measurement floor that is 31–45 μm [24]. The floor effect is obtained due to architectural support of IPL. Macular Cube 512×128 or 200×200 scan protocol and automated GC‐IPL analysis segmentation algorithm and optic disc 200×200 protocol incorporated into the Cirrus 6.0 software were used. The average GC-IPL and pRNFL thicknesses were studied and groups compared.

### Visual field assessment

Visual fields were assessed with the Humphrey Field Analyzer perimetry as previously described [18]. The VFI is a single number that summarizes each patient’s visual field status as a percentage of the normal age-corrected sensitivity. It is approximately 100% in normal fields and approaches 0% in perimetrically blind fields. It provides improved correspondence to ganglion cell loss compared to mean deviation [25].

### Statistics

Descriptive statistics are presented as means and standard deviations (SD). T-test was used to report statistical difference between age at OPG diagnosis among children with NF1-associated OPG and sporadic OPG, age difference between first reliable VA and VF examination and between first reliable OCT examinations. The Generalized Linear Mixed Models (GLMM) of statistical package SPSS (ver.26) was used to compare the groups. The dependent variables were the VA, GC-IPL, pRNFL and VFI. The fixed factors were OPG group, age, the random factor was the examination time. Pairwise contrasts test was used to evaluate the difference between the NF1-OPG and NF1 noOPG groups. The follow-up analyses are made using a repeated measure correlation (rmc) script in R to evaluate for each subject the regression line of each variable during the follow-up time. The estimated means, provided by the GLMM represent estimates of the studied parameters. In addition, we have compared VA and GC-IPL results in children with NF1 without symptomatic OPG with previous studies on healthy typically developed children <18 years old [17,26].

## Results

### Patients

Sixty-eight of the 81 (84%) invited patients with NF1 diagnosis accepted the invitation. Thirteen patients without symptomatic OPG choose not to participate in the study. None of the 16 children with NF1 and MRI-verified OPG declined participation. An additional 9 patients with S- OPG were invited and all 9 accepted to participate. Thus, a total of 77 patients (46 boys and 31 girls) were included in the study, of which 25 had OPG (32%). Of the 68 children with NF1 diagnosis 63% had neuropsychiatric disorders (cognitive deficits, specific learning problems, attention deficit hyperactivity disorder or autism spectrum disorders). Both eyes were affected by the OPG in 11 out of 16 patients (69%) with NF1 and in 6 out of 9 (67%) with S-OPG. Children with S- OPG were diagnosed with OPG earlier (1.8±2.5 yrs) than children with NF1 (4.3±2.8 yrs) (p<0.001). There was no difference in age between those with and without OPG (p>0.05) at the study start. At study start there were three children who were blind in both eyes (one with NF1-associated OPG with NLP in both eyes, one with S-OPG and NLP in both eyes and one with S-OPG and CF in both eyes), two NF1 patients with moderate VI and 72 patients without VI. In total there were 11 blind eyes (four NF1-related and seven sporadic). Mean age for first reliable VA measurement was significantly lower than for VFI (5.4±1.6 yrs vs 7.3±1.0 yrs; p<0.001) and for first reliable GC-IPL measurement compared to pRNFL (5.6±1.5 vs 6.8±1.3; p<0.001). Table 1 presents mean age (±SD; range) between groups of the patients at the first available examination in at least one eye. Magnetic resonance imaging was performed due to ophthalmological symptoms in 14 cases (eight NF1-OPG, six S-OPG) and neurological and/or endocrinological symptoms in 11 cases (eight NF1-OPG, three S-OPG).

**Table 1:** Age at first reliable examination among patients in different groups.

	NF1 without OPG (N = 52)	NF1 with OPG (N = 16)	Sporadic OPG (N = 9)
**Gender (M/F)**	34/18	7/9	5/4
**VA mean (SD; range) yrs**	5.2 (± 1.5; 3.0–9.0)	5.5 (± 1.7; 3.0–8.4)	6.0 (± 2.4; 3.0–10.0)
**GC-IPL mean (SD; range) yrs**	5.5 (± 1.4; 3.0–9.0)	6.0 (± 1.3; 4.1–8.4)	6.0 (± 1.5; 3.5–7.8)
**pRNFL mean (SD; range) yrs**	6.8 (± 1.3; 4.0–9.3)	7.4 (± 1.5; 5.6–9.1)	7.4 (± 0.3; 7.0–7.8)
**VFI mean (SD; range) yrs**	7.2 (± 1.0; 5.2–9.9)	7.0 (± 0.8; 6.0–7.7)	7.3 (± 0.8; 6.3–8.2)

NF1—neurofibromatosis type 1, OPG—optic pathway glioma, M-male, F-female, VA—visual acuity, SD- standard deviation, yrs-years, GC—IPL—ganglion cell—inner plexiform layer, pRNFL- peripapillary retinal nerve fiber layer, VFI—Visual Field Index.

**Fig 1:**
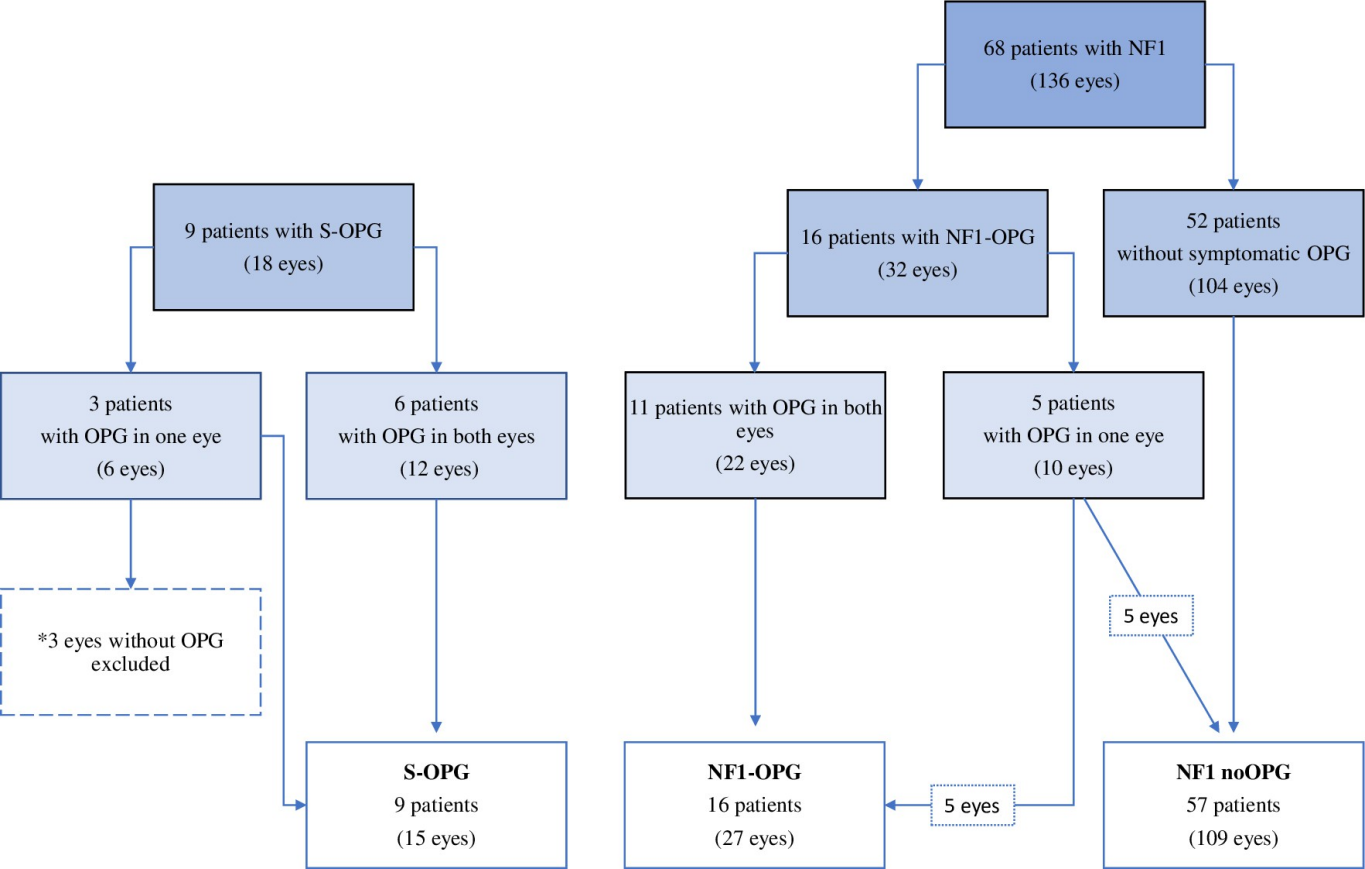
Flow chart of study population. NF1—neurofibromatosis type 1, OPG—optic pathway glioma, S-sporadic.

There were 154 eyes examined during the study period (Fig 1). Three unaffected eyes from patients with sporadic isolated optic nerve glioma were excluded, as explained above. There were 109 eyes in NF1 noOPG group, 27 eyes in NF1-OPG group and 15 eyes in S-OPG group. All 151 eyes provided VA data, while GC-IPL data were obtained in 132 eyes (Complete scans 88%) from 71 patients, pRNFL in 73 (49%) eyes from 43 patients and VFI measurements in 55 eyes (37%) from 36 patients at least once during the study period. In total, GC-IPL was obtained in 95% eyes without symptomatic OPG and in 69% eyes with OPG, pRNFL in 54% eyes without symptomatic OPG and in 33% with OPG and VFI in 39% of eyes without symptomatic OPG and in 31% eyes with OPG. The remaining eyes did not provide GC-IPL scans due to blindness (27% of eyes in the group with OPG) or provided unreliable scans due to artifacts (5% in the group without symptomatic OPG; 4% in the group with OPG).

Table 2 lists mean values, SE and 95% CI for all four parameters in all groups. Pairwise contrast tests showed thinner OCT measurements (GC-IPL and pRNFL; p<0.001), lower VA (p<0.001) and VFI (p = 0.022) in eyes in group NF1-OPG compared to eyes in group NF1 noOPG. The GC-IPL was significantly correlated to pRNFL (correlation coefficient (r) = 0.662, confidence interval (CI) = .507 to .773 p<0.001), VA (r = -0.661, CI = -7.45 to -.551, p<0.001) and VFI (r = 0.644, CI = .452 to .774, p<0.001).

**Table 2:** Comparison of estimated values of four parameters (VA, VFI, GC-IPL and pRNFL) among three groups of eyes.

	NF1 noOPG N = 109	NF1 -OPG N = 27	S- OPG N = 15	p
**VA logMAR, mean ± SE (95CI)** **N = eyes**	0.08 ± 0.1 (0.05–0.1) N = 109	0.19 ± 0.2 (0.16–0.23) N = 27	1.02 ± 0.1 (0.87–1.16) N = 15	<0.001
**VFI, mean ± SE (95CI) %** **N = eyes**	95 ± 0.6 (94–97) N = 42	88 ± 3.0 (83–94) N = 9	47 ± 1.8 (43–50) N = 4	<0.001
**GC-IPL, mean ± SE (95CI) μm** **N = eyes**	85 ± 0.4 (84–85) N = 103	70 ± 0.7 (68–70) N = 22	62 ± 1.4 (60–65) N = 7	<0.001
**pRNFL, mean ± SE (95CI) μm** **N = eyes**	100 ± 0.9 (98–101) N = 59	70 ± 2.1 (66–74) N = 11	77 ± 2.6 (72–83) N = 3	<0.001

VA—visual acuity, VFI- Visual Field Index, GC-IPL—ganglion cell—inner plexiform layer, pRNFL- peripapillary retinal nerve fiber layer, NF1—neurofibromatosis type 1, OPG—optic pathway glioma, S—sporadic, SE—standard error, CI—confidence interval.

**Fig 2:**
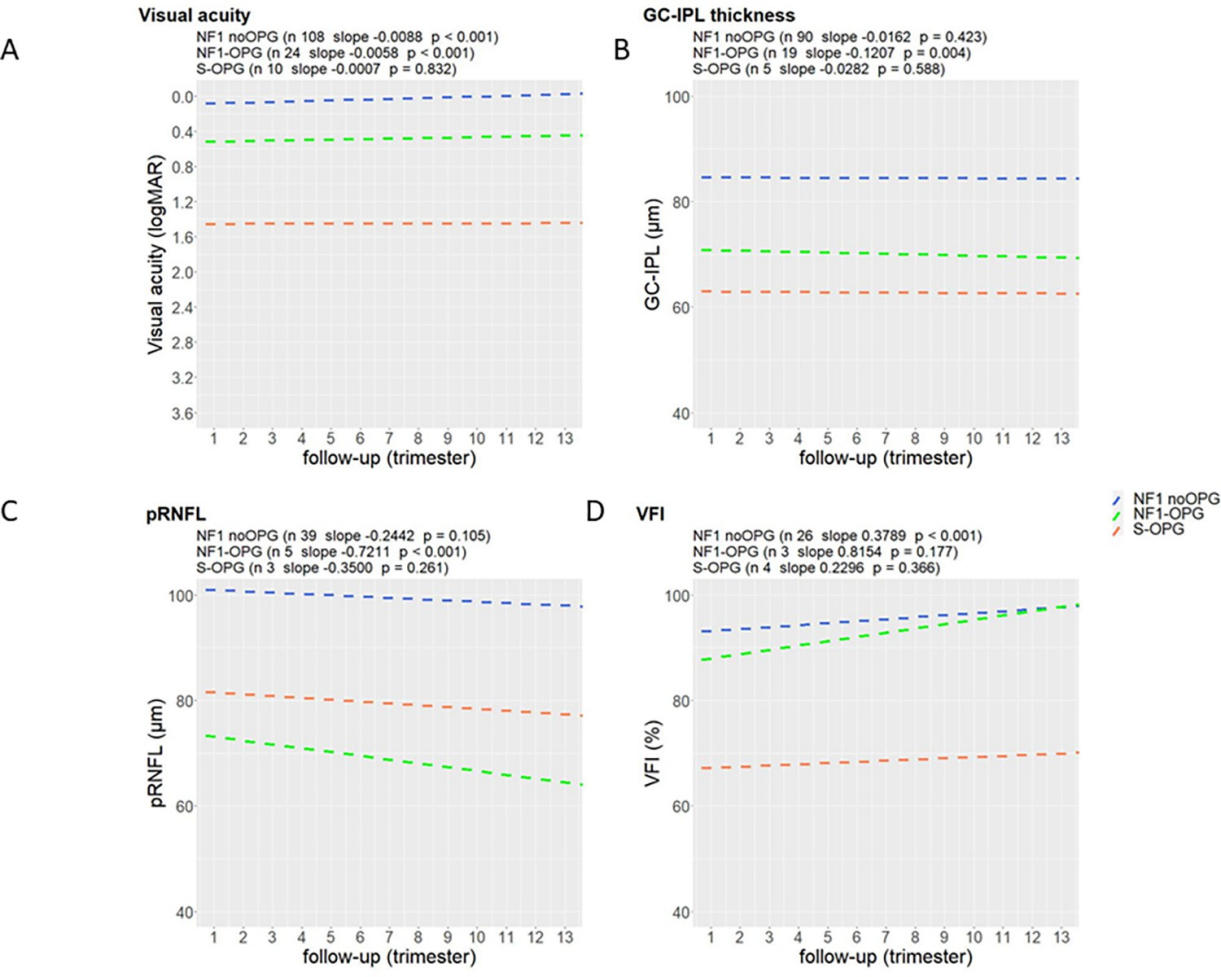
A model of change in four main parameters during a three-year study period in the groups. A) VA, B) GC-IPL, C) pRNFL, D) VFI. NF1—neurofibromatosis type 1, OPG—optic pathway glioma, VA- visual acuity, GC-IPL—ganglion cell—inner plexiform layer, pRNFL- peripapillary retinal nerve fiber layer, S—sporadic, VFI- Visual Field Index.

Fig 2 presents change over time of VA, GC-IPL, pRNFL and VFI for the subjects of each group. All eyes needed to provide at least two measurements to be included in the graph, thus conferring a reduction in number of eyes analyzed specifically regarding pRNFL and VFI in NF1-OPG and S-OPG groups. All eyes provided VA measurements on at least two occasions, except initially blind children (that had only one examination during the study period). Repeated Measures Correlation analysis showed that VA was slightly but significantly improving over time in NF1 noOPG eyes (slope -0.0088/trimester, p<0.001) and in NF1-OPG eyes (slope -0.0058/trimester, p<0.001). The S-OPG group eyes demonstrated consistently low vision across the study period without statistically significant improvement (p = 0.0832). In general, both GC-IPL and pRNFL were reduced at the study entry in OPG eyes. A slight but significant decline in GC-IPL was observed in the NF1-OPG group (slope -0.1207μm/trimester, p = 0.004) and insignificant changes were seen in the remaining groups (p>0.05). All three groups showed a thinning in pRNFL over time, however reaching statistical significance in NF1-OPG group (slope -0.7211μm/trimester, p<0.001). Visual field index was improving in all groups but significant results were obtained only in the NF1 noOPG group (slope 0.3789%/trimester, p<0.001).

### Patients with OPG

**Fig 3:**
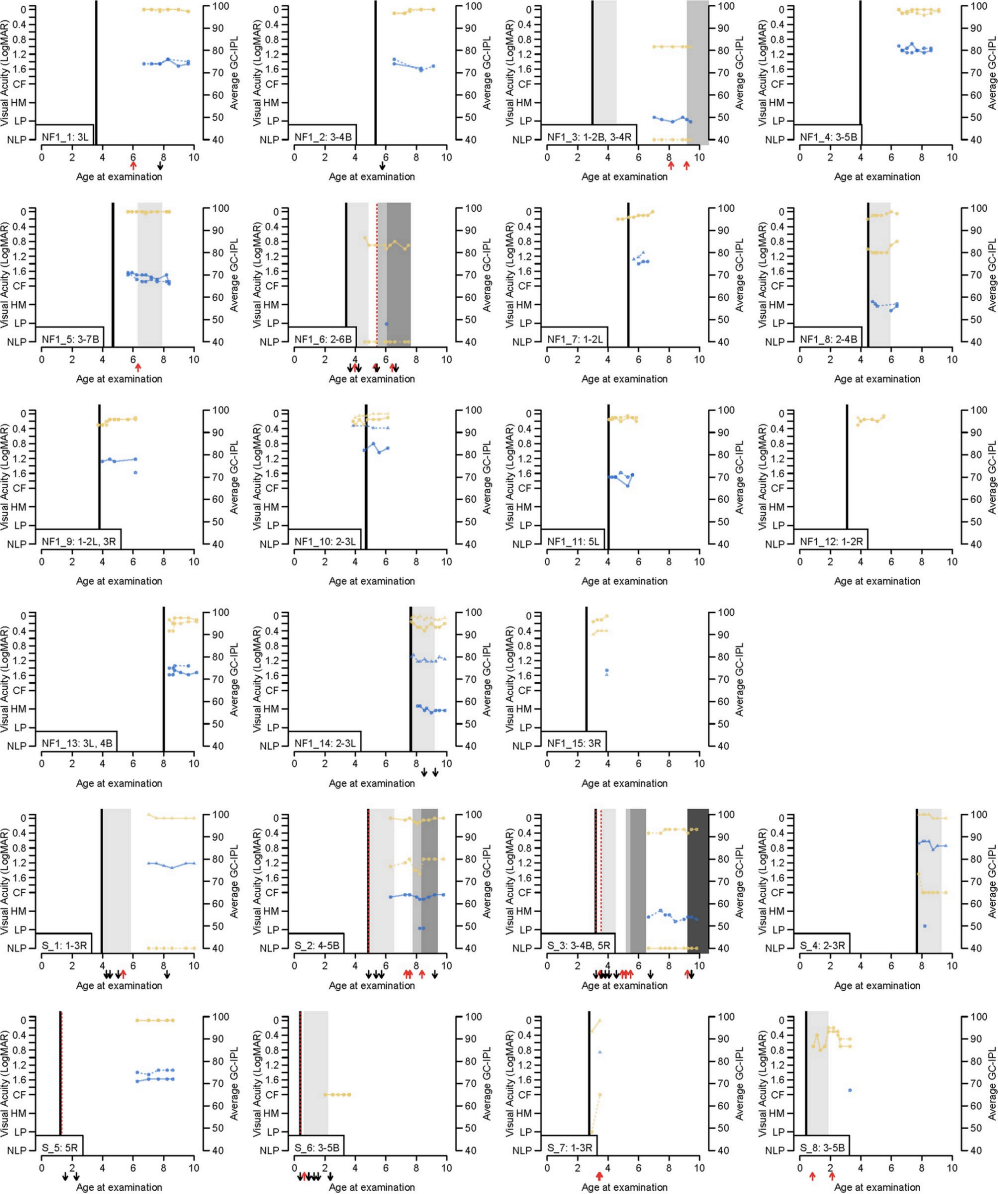
Graphs present longitudinal VA and GC-IPL measurements obtained during the study period. CF: count fingers (LogMAR = 2.0); GC-IPL: ganglion cell inner plexiform layer; HM: hand movement (LogMAR = 2.5); LE: left eye; LP: light perception (LogMAR = 3.0); NF1: neurofibromatosis type 1; NLP: no light perception (LogMAR = 3.5); RE: right eye; S: sporadic optic pathway glioma. Different anatomic locations of optic pathway gliomas. Extension of tumors: (1) intraconal (posterior boundary orbital apex); (2) intracanalicular (posterior boundary optic foramen); (3) intra‐cranial‐prechiasmatic; (4) chiasmatic; (5) optic tract; (6) lateral geniculate nucleus; (7) optic radiation; B: both left och right, L: left; R = right.

Fig 3 presents the individual clinical follow-up records of all patients with OPG, except for two blind patients. At least two measurements per eye were required (VA or GC-IPL) for the eye to be included in the graph. Seven patients showed radiological progression (MRI) during the study period (NF1_3, NF1_5, NF1_6, S_2, S_3, S_7, S_8). Out of these, four showed parallel GC-IPL thinning (NF1_3, NF1_5, S_2, S_3). In the remaining three cases the thinning could not be assessed due to no or only one GC-IPL measurement. Case NF1_6 was blind in one eye and the other eye had low VA with one measurement of average GC-IPL at 48 μm. Case S_7 with optic nerve glioma in one eye and CF VA in the eye with glioma did not provide GC-IPL measurements. Case S_8 had low VA in both eyes and GC-IPL in one eye at 67 μm. The results showed larger intraindividual differences in VA measurement than in GC-IPL during the study period over time. Graphs shows that when VA was impaired, GC-IPL results were consistently subnormal. It was observed that GC-IPL in eyes with OPG remained low while VA may actually have improved over time. In two cases average GC-IPL thickness was above 76 μm (NF1_4 and NF1_10) and VA was near normal (-0.1 logMAR).

Mean age at time of first line treatment was 3.8±2.5 yrs (six girls, seven boys). Thirteen patients (52%) (six with NF1-OPG, seven with sporadic OPG) received chemotherapy, of which ten patients because of ophthalmological symptoms. Ten patients received vincristine as first line chemotherapy according to the Low-Grade Gliomas protocol [23]. Three patients (one NF1-OPG, two S-OPG) were started on vinblastine because of very young age according to recommendations [27]. Adverse effects of carboplatin resulted in treatment switch to vinblastine in seven patients. Subsequent second- or third-line therapy was applied in six cases due to tumor progression. Five patients received at least one additional bevacizumab treatment under ongoing treatment. Six patients underwent surgery at least once. One patient underwent only surgery without subsequent chemotherapy, whereas five patients had initial surgery due to hydrocephalus, tumor size or epilepsy.

## Discussion

This three-year follow-up study assessed the ophthalmological functional and morphological changes in children with NF1 with and without symptomatic OPG as well as in children with sporadic OPG. We showed that OCT in general and GC-IPL in particular was a reliable method that could confirm symptomatic OPG along the optic pathway. In addition, this study demonstrated significant correlations between GC-IPL and pRNFL, VA and VF measurements.

We found that eyes in children with NF1 without symptomatic OPG had thicker GC-IPL and pRNFL than eyes in children with OPG and NF1. The estimated mean GC-IPL values in both OPG groups were below the fifth percentile of the healthy population of children as presented in our earlier study, in contrast to average GC-IPL values for eyes without symptomatic OPG that fell into the normal range of healthy eyes (>fifth percentile) [17,26]. Thus, the GC-IPL reduction was not associated with NF1 diagnosis per se but with the presence of OPG. However, it cannot be ruled out that conditions secondary to OPG may have contributed to the damage, such as e.g. hydrocephalus.

Our study showed that the estimated mean pRNFL for all eyes with OPG was significantly lower than in eyes without symptomatic OPG, similarly to previous reports [11]. We found a significant correlation between the GC-IPL and pRNFL (r = 0.662, p<0.001). The significant correlation, as previously demonstrated, assumes that they are interchangeable [28]. In general, one of the advantages of GC-IPL compared to pRNFL measurements is that the GC-IPL layers do not swell and do not show false increase in thickness. Also, pRNFL has shown larger inter-subject variability, (due to effects of peripapillary blood vessels and refractive errors) compared to GC-IPL [29].

This study showed that eyes in children with NF1 without symptomatic OPG had better VA and higher VFI than eyes in children with NF1 and MRI- verified OPG. Visual acuity is less sensitive in young children due to different rates of maturation. In that regard there is a need of objective measures that reduce the risk of bias. Thinning of GC-IPL can then strengthen the clinical value of low VA in children otherwise interpreted as unconcentrated. While differing VA between visits might be the result of poor participation, on the other hand a lack of normal age-dependent improvement may in fact mask an underlying deterioration. This study showed a significant correlation between GC-IPL and visual function (p<0.001). This strong correlation supports the hypothesis that the average GC-IPL thickness can detect the loss of ganglion cells with the eyes with OPG. This is in accordance with study by *Gu et al* who found that GC-IPL significantly decreased in children with OPG-related vision loss compared to those with normal vision [14]. A significant strong correlation between the thinning of GC-IPL and the VF loss was due to the fact that at the macula, the bodies of the neurons are more numerous and topographically organized to correspond to the VF. This may be especially useful in patients unable to cooperate with functional testing. It may also serve as an additional measure to demarcate VF defects and as predictor of a permanent vision defect as described in previous study [18].

One interesting finding was that a subnormal GC-IPL in the setting of normal VA in many cases was shown to be an early sign of tumor involvement (Fig 3). *Nujits et al*. found that children with OPG and normal visual function have greater macular GC- IPL thickness (median 76 μm, range, 61–87 μm) compared to those with abnormal visual function [15]. In other words, in eyes with tumor progression affecting the visual pathway, thinning of GC-IPL may precede and indicate future functional impairment. Children with normal GC-IPL thickness may require a larger decline to express visual loss compared to children with thinner baseline GC-IPL thickness. There were only three eyes with OPG (Fig 3, NF1_4 and NF1_10) that showed average GC-IPL thickness above 76 μm, that is above fifth percentile of average GC-IPL thickness in healthy children [17], despite presence of OPG. In the NF_10 case OPG was located in left optic nerve with VA -0,10 logMAR and GC-IPL above the 76 μm in that eye and VA 0.0 logMAR and average GC-IPL 90 μm in the corresponding eye. Thus, in case of difference in VA and GC-IPL between the eyes underlying pathology could be suspected despite presumed normal average GC-IPL thickness.

This study showed that despite the presence of OPG, eyes showed increasing VA in the NF1-OPG group during the study period (Fig 2A). This was not seen in the sporadic OPG group, possibly since these eyes entered the study with a significantly lower VA and thus less neuronal reserve. It seems as in the NF1-OPG group, the treatment regimen prevented further visual deterioration and enabled visual maturation, however at different level compared to eyes without symptomatic OPG. Regarding GC-IPL thickness in the macula, both OPG groups underwent thinning over time at different rates, however, the change reached statistical significance only in the NF1-OPG group (Fig 2B). However, there were only five eyes were included in the sporadic group, so the lack of significance must be interpreted with caution. There was a slight but progressive pRNFL decrease during follow-up reaching statistical significance only in the NF1-OPG group (Fig 2C). One possible explanation is that, despite treatment, axonal degeneration secondary to tumor compression still proceeds in parallel to the course of the disease constituting the basis for additional long-term optic neuropathy [8]. The visual fields showed improvement (VFI) over time in all groups (Fig 2D) which was assumed to be a learning on the repeated testing.

In cases where there is an obvious and progressive GC-IPL loss, but normal or near normal VA, one can whether delaying treatment means an increased risk of vision loss. *Gu et al*. mentions this progressive GC-IPL loss as a potential indication for treatment in order to prevent decrease in VA [14]. Indeed, the goal of treatment is precisely to prevent vision loss. A presence of new or progressive VA decline was the most frequent indication to initiate therapy in both the NF1 and sporadic groups (79%). Sometimes progressive VA decrease could have been seen despite the ongoing treatment (e.g. Fig 3. S_2). In these cases, the confirmation of further GC-IPL reduction supported a change in treatment.

The prevalence of OPGs detected by MRI in our pediatric NF1 population was 23%, which is slightly higher than in other studies 15–21% [5,30]. But in the dropout group there were only patients with NF1 who had no symptoms of OPG. If one includes these in the frequency analysis, the proportion of cases is on par with previous studies (16/81; 20%). In this study children with NF1- OPG were diagnosed with OPG approximately at four years of age. Patients with sporadic OPG were significantly younger at diagnosis of OPG than patients with NF1. This is in accordance with a previous study [31]. According to literature, the mean age at diagnosis of OPG in NF1 patients varies between three to six years of age [4,32–34], with estimates in the lower part of this range in studies with MRI screening for OPG [5,34]. One might think that the screening of NF1 children should mean that the diagnosis of OPG should come earlier in this group compared to sporadic OPGs. However, there was a trend in our study that the eyes in the S-OPG group had worse results than the eyes in the NF1-OPG group right from the start of the study. This disadvantage of the S-OPG group can be interpreted as that only the most severe cases of sporadic OPG were detected early and only after clear symptoms had been observed by parents, whereas the systematic ophthalmological screening of children with NF1 meant that tumors with more moderate visual impairment were detected [35,36]. Finally, our study showed that GC-IPL measurements were easy to obtain and at similar early age as VA and considerably earlier than both VF examinations and reliable pRNFL measurements. This is particularly important in this clinical group who are at risk of developing OPG at an early age. Obtaining reliable pRNFL was more challenging than GC-IPL thickness images due to increased frequency of poor image focus, off-center circumpapillary circle and varying position of the superior and inferior vascular arcades that falsely elevated or reduced the thickness measures. The missing GC-IPL was due to blindness and artifacts. Visual field testing was not possible or not reliable for most of the children, mostly due to young age, reduced cooperation or general clinical condition of the patient. Acquiring reliable measurements in this patient group is particularly challenging since many patients with NF1 have coexisting neuropsychiatric disorders. As mentioned above, 63% of NF1 patients in this study had cognitive deficits, specific learning problems, attention deficit hyperactivity disorder or autism spectrum disorders. According to previous studies 80% of patients with NF1 have been shown to exhibit cognitive deficits, 30–65% learning problems, as well as 30–50% meeting criteria of attention deficit hyperactivity disorder [8].

### Strengths and limitations

The strengths of the present study include standardized assessment of ocular factors. Although the study period may be considered relatively short, it provides a continuous registration under standardized conditions of different ophthalmological outcomes and enables comparisons between these in terms of reliability and diagnostic value. Our study had some limitations. Children with NF1 without symptomatic OPG did not undergo MRI. In our clinic, screening with MRI is not systematically performed in all patients. Routinely, MRI is only performed in cases where clinical findings are suggestive of OPG. However, the ophthalmic outcomes in the NF1 noOPG group, including VA and GC-IPL were similar to previous findings in healthy subjects. Since they were asymptomatic there is no indication either for MRI or for treatment.

## Conclusions

The macular GC-IPL as measured with OCT distinguished well between eyes with MRI-verifed OPG and eyes without symptomatic OPG in children with NF1 and correlated well with visual function. Unlike VA, GC-IPL is not affected by age in young children, rendering GC-IPL measurements more reliable in the follow-up of OPG. Low VA in children can be misinterpreted, thus thin GC-IPL can readily increase the reliability of the examination in order to prevent delay of neuroradiological testing. We recommend to include macular GC-IPL analysis for screening purposes in children with NF1. Considering the occurrence of S-OPG we also recommend to include GC-IPL measurements in all children with subnormal VA or poor performance in VA assessments.

## Abbreviations

CI: confidence interval
CF: counting fingers
GC-IPL: ganglion cell inner plexiform layer
HM: hand movement
LP: light perception
MRI: magnetic resonance imaging
NF1: neurofibromatosis type 1
NLP: No light perception
OCT: optical coherence tomography
OPG: optic pathway glioma
pRNFL: peripapillary nerve fiber layer
S: sporadic
SD: standard deviation
SE: standard error
VA: visual acuity
VI: visual impairment
VF: visual field
VFI: visual field index
yrs: years
